# Target-Based Whole-Cell Screening by ^1^H NMR Spectroscopy**

**DOI:** 10.1002/anie.201410701

**Published:** 2015-02-18

**Authors:** Junhe Ma, Qing Cao, Sarah M McLeod, Keith Ferguson, Ning Gao, Alexander L Breeze, Jun Hu

**Affiliations:** Discovery Sciences, AstraZeneca R&D BostonWaltham, Massachusetts 02451 (USA); Infection Innovative Medicines, AstraZeneca R&D BostonWaltham, Massachusetts 02451 (USA); Discovery Sciences, AstraZeneca R&D, Alderley ParkMacclesfield, Cheshire, SK10 4TG (UK); Astbury Centre for Structural Molecular Biology, Faculty of Biological Sciences, University of LeedsLeeds, LS2 9JT (UK)

**Keywords:** drug discovery, in vivo methods, New Delhi metallo-β-lactamase, NMR spectroscopy, whole-cell screening

## Abstract

An NMR-based approach marries the two traditional screening technologies (phenotypic and target-based screening) to find compounds inhibiting a specific enzymatic reaction in bacterial cells. Building on a previous study in which it was demonstrated that hydrolytic decomposition of meropenem in living *Escherichia coli* cells carrying New Delhi metallo-β-lactamase subclass 1 (NDM-1) can be monitored in real time by NMR spectroscopy, we designed a cell-based NMR screening platform. A strong NDM-1 inhibitor was identified with cellular IC_50_ of 0.51 μm, which is over 300-fold more potent than captopril, a known NDM-1 inhibitor. This new screening approach has great potential to be applied to targets in other cell types, such as mammalian cells, and to targets that are only stable or functionally competent in the cellular environment.

Drug discovery often begins with screening a collection of compounds in the hope that some active molecules can be identified and later developed into drugs.[1] Phenotypic screens, which seek compounds that induce a change of phenotype in cells, tissues or organisms, were the mainstay of the drug discovery process before the genomics age.[2] Advances in molecular biology, genomics, structural biology, and computational modeling subsequently shifted the search for drugs in favor of target-based approaches.[3] Both screening methods have their own advantages and disadvantages.[1], [4] For phenotypic screens, a major hurdle is understanding the mechanism of action behind screening hits, because identification of the target(s) is critical for further optimization of compounds showing phenotypic interference. Target-based screening, however, employs highly simplified systems in which targets are isolated from their complex biological networks. Classical in vitro biochemistry and biophysics approaches are powerful methods for hit identification and verification, and structural biology plays a key role in guiding chemists to design specific and potent inhibitors. Nevertheless, eventually those compounds showing activities in in vitro assays must prove to possess activity in vivo as well. Very often inhibitors fail at this stage owing to unfavorable physicochemical properties, efflux, and poor cell penetration.

The concept of “target-based” whole cell screening has been introduced recently to overcome some of these limitations whilst retaining the merits of both of these screening procedures. Approaches exploiting antisense silencing technology or arrays of genetically-altered bacterial strains with inducible control of target expression have been used to screen for and identify inhibitors with antibacterial activity against pathogenic organisms.[5]–[7] However, these approaches may still be susceptible to off-target-mediated effects, giving rise to false positives. Hence, there is a need for new screening methods that combine the rigor of in vitro target-based approaches with the biological context implicit in phenotypic screens.

Recently, we reported that the hydrolysis of meropenem by *E.* *coli* cells carrying New Delhi metallo-β-lactamase subclass 1 (NDM-1) can be readily monitored by ^1^H nuclear magnetic resonance spectroscopy (NMR).[8] This finding has led us to design an NMR-based whole cell screening experiment that utilizes ^1^H NMR signals from meropenem (the substrate) and hydrolyzed meropenem (the product) as end points to detect enzyme inhibition in the native bacterial cell context (Figure 1).

**Figure 1:**
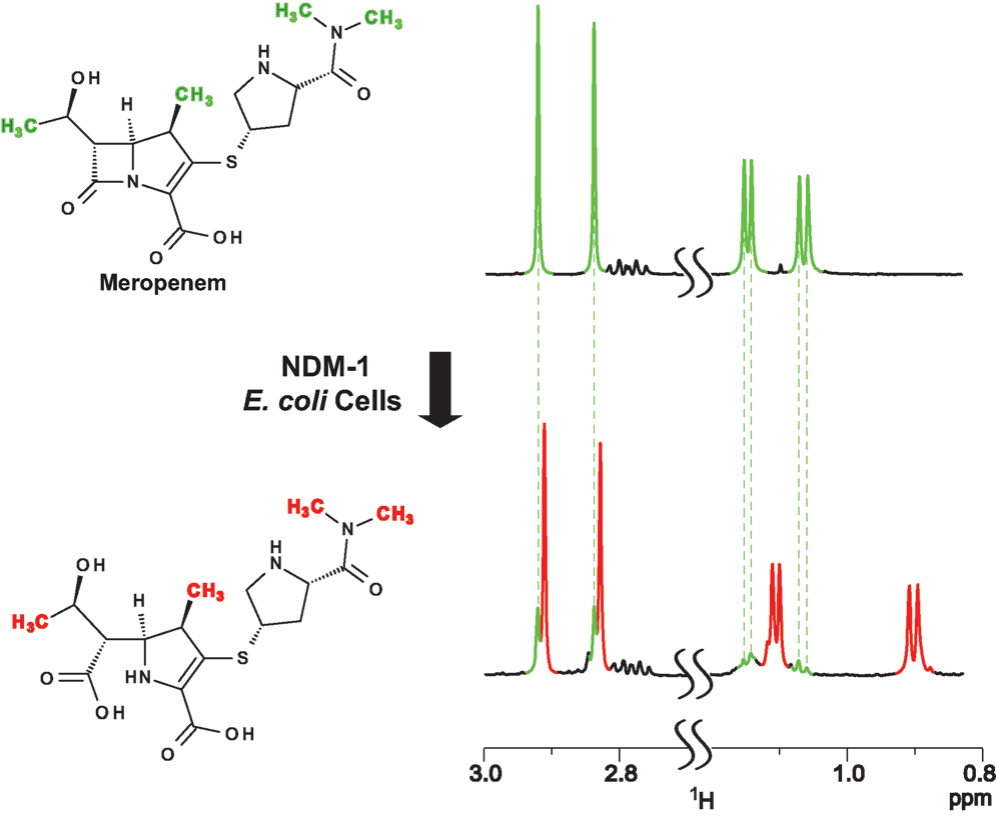
^1^H NMR spectra of meropenem hydrolysis catalyzed by NDM-1 *E.* *coli* cells. Only ^1^H signals of methyl groups are shown. Signals from meropenem and the hydrolyzed product are colored in green and red, respectively.

Bacteria produce a variety of β-lactamases that defend them from attack by β-lactam antibiotics.[9] NDM-1 is a Class B or metallo-β-lactamase.[9], [10] In contrast to Class A, C, and D β-lactamases that exploit an active site serine residue to catalyze ring opening of β-lactams, Class B β-lactamases utilize bound Zn^2+^ ions to mediate hydrolysis. The recently discovered NDM-1 strains pose a potential worldwide epidemic threat as bacteria carrying this enzyme may be rapidly disseminated across continents and become resistant to the majority of antibiotics including carbapenems, considered one of the last lines of defense against serious bacterial infection.[11] Despite the urgent medical need, little advance has been made in the development of clinically useful NDM-1 inhibitors.[9a, 11b]

To implement a 96-well plate format to screen NDM-1 inhibitors, we selected 92 compounds that are either known metallo-β-lactamase inhibitors or derivatives of them.[10] The first and last two wells act as controls and are filled with deuterated dimethyl sulfoxide ([D_6_]DMSO) and ethylenediaminetetraacetic acid (EDTA). All compounds are dissolved in [D_6_]DMSO to give a final stock concentration of 100 mm. The screening experiment comprises three simple steps (Figure 2): 1) compound incubation with NDM-1 *E.* *coli* cells; 2) addition of meropenem; and 3) NDM1-catalyzed hydrolysis reaction, quenched by addition of 10 mm EDTA. The final concentrations for NMR detection are 500 μm compound, 100 μm meropenem, 0.5 % [D_6_]DMSO, and 10 mm EDTA. Under the conditions prior to the addition of 10 mm EDTA, the cells appear to be healthy (Supporting Information, Figure S2). The experimental procedure of screening setup is detailed in the Supporting Information. After quenching the reaction, the plate was transferred to an automated NMR screening system to acquire 1D ^1^H NMR spectra of each well on the plate.

**Figure 2:**
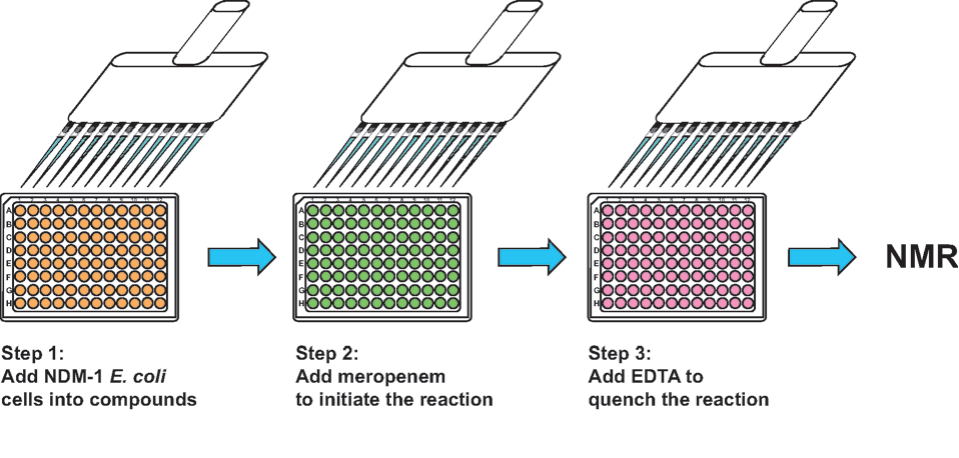
Stepwise preparation of a target-based whole-cell screening plate for NMR data acquisition.

Figure 3 presents the 0.85–0.95 ppm region of ^1^H NMR spectra from each well in a 96-well plate format. For compounds with signals overlapping with the 0.9 ppm signal (for example, well A10 and G10 signals in Figure 3), methyl ^1^H signals around 1.1 and 2.9 ppm area can be examined for further validation (Supporting Information, Figure S1). In Figure 3, signals of well A1 and H12 were blank controls in which only [D_6_]DMSO was added. Examination of signals at 1.1 ppm indicates that meropenem was completely hydrolyzed. Signals of well A2 and H11 were from samples with 500 μm EDTA. Since EDTA is a potent inhibitor of NDM-1 in *E.* *coli* cells (the fifty percent inhibitory concentration, IC_50_, is 1.6 μm),[8] hardly any product signal is detectable. Thus, these two wells represent positive controls for strong NDM-1 inhibitors.

**Figure 3:**
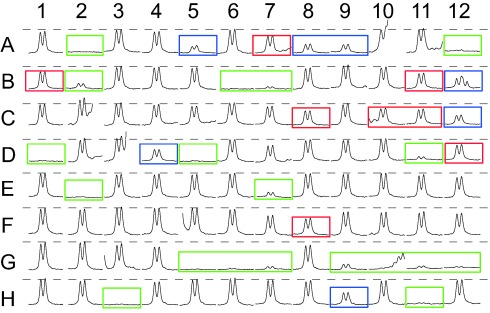
NMR screening results shown in a 96-well plate format. The product signal at 0.9 ppm was used to monitor the inhibition. Weak, medium, and strong inhibitions are framed in red, blue and green boxes, respectively.

The potency of screening hits was ranked as strong (>80 %), medium (50–80 %), and weak (20–50 %) based on the percentage inhibition (Supporting Information, Table S1). As most compounds in the 96-well plate are either known Class B β-lactamase inhibitors or their derivatives, it is not surprising that a high hit rate of 34 % was observed. Many compounds showed potencies similar to EDTA. We then determined by NMR spectroscopy the IC_50_ values against NDM-1 enzymatic activity in bacterial cells of three screening hits (C10, A5, and B6) that were ranked as weak, medium, and strong, following our previously reported procedure (Figure 4).[8] The strong and weak inhibitors from our NMR-based screen yielded IC_50_ values of 0.51 and 120 μm, similar to those of EDTA and L-captopril (IC_50_=175 μm), respectively.[8] Although only three compounds are shown here, an initial structure–activity relationship emerges from the analysis of the chemical structures. All three compounds possess a similar captopril-like scaffold. Evidently, the carboxylate group on the right hand side of compound B6 and bulky aromatic groups play important roles in potency. An X-ray crystallography study of NDM-1/B6 complex structure is currently ongoing to investigate the key interactions contributing to the potency.

**Figure 4:**
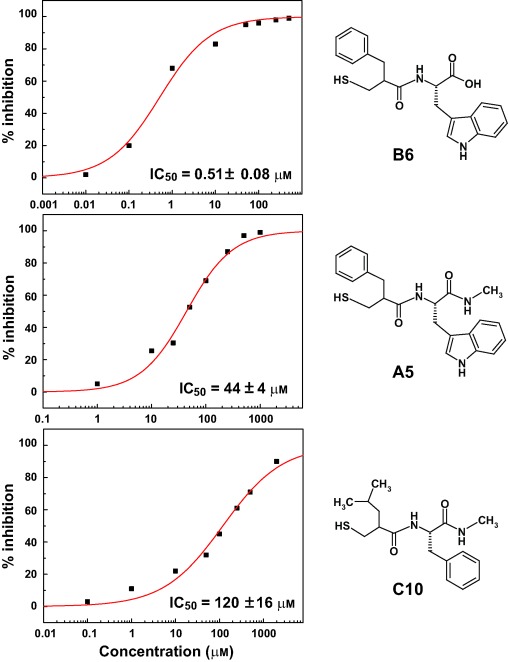
IC_50_ measurement of selected NDM-1 inhibitors based on their potency rank from the NMR screen. A5, B6, and C10 are the coordinates of samples in the 96-well plate shown in Figure 3. The chemical structures of the compounds selected for IC_50_ measurement are shown on the right side of the fitting curve. The procedure for measurement of IC_50_ is detailed in the Supporting Information.

Using the same setup, we evaluated 92 hits from a conventional high-throughput phenotypic antibacterial screen of one million compounds run against the NDM-1 *E.* *coli* cells. Surprisingly, only 18 % of the hits from this screen were confirmed to inhibit meropenem hydrolysis in our whole-cell NMR assay (data not shown). This illustrates clearly the ability of the whole cell-based NMR assay to directly detect hits by the desired mechanism of action, without the onerous requirement for additional assays to eliminate false positive hits, and further underlines the potential for our method to be integrated into drug discovery programs.

Although NMR spectroscopy is commonly applied in vitro to screen compound (and especially fragment) libraries against certain targets,[12] to the best of our knowledge NMR spectroscopy has not been applied to date for the screening of endogenous enzymes in whole cell samples. The setup is straightforward and robust as long as a specific enzymatic reaction or target binding is monitored and the solubility of the probed substrate is over 50 μm. In contrast to other biophysical methods such as surface plasmon resonance and mass spectrometry, no immobilization of cells or post-screen cell lysis are required. It is cost-effective and time-efficient as protein over-expression and purification steps are avoided. It takes approximately 5 min to record data for one sample. If a mixture of 5 compounds were screened instead of the singlets as shown here, the throughput would potentially be over 10 000 compounds per week. Furthermore, based on coarse ranking of compound potency, hits can be followed up by cellular IC_50_ measurement using NMR spectroscopy, which can also be handled in an automated fashion.

Besides β-lactamases in bacteria, many other targets should be amenable to this approach. For example, further to monitoring enzymatic reactions, several types of binding interactions on cellular surfaces, such as those of small protein growth factors, hormones, or neurotransmitters to their receptors on cancer, endocrine, or neuronal cells, should be observable by NMR spectroscopy. It should in principle be straightforward to design NMR screens aiming to find molecules disrupting these interactions. As well as ^1^H, other nuclei such as ^13^P, ^19^F, and ^13^C can also be used as NMR probes for screening.

In summary, we have developed a novel NMR screening method for discovering inhibitors against a specific target in cells where a diffusible substrate or product can be monitored. This approach enjoys the merits of both target-based and whole-cell screening approaches. The outcome of the screen is more predictive of a biologically relevant effect because targets reside in the physiological environment in which enzymatic reactions and inhibition occur.[8] The laborious steps of target identification in traditional whole-cell screening are rendered unnecessary, and the gap in potency between in vitro and in vivo assays is bridged as compound permeability, efflux pumps, and target accessibility are intrinsically factored in. This method can be applied to challenging proteins (for example, membrane proteins) that are hard to obtain in purified, active form for in vitro assays. We believe that in principle this type of NMR screen has the potential to be adapted for use against other types of biological samples such as cancer cells, blood cells, and viral particles.

## Notes

Supporting information for this article (including the Experimental details) is available on the WWW under http://dx.doi.org/10.1002/anie.201410701.
