# Supplementary material for: Target-Based Whole-Cell Screening by 1H NMR Spectroscopy
Source: Angew Chem Int Ed Engl. 2015 Feb 18;54(16):4764–7. doi: 10.1002/anie.201410701 (PMC4471574; doi:10.1002/anie.201410701)
Supplement: Supplementary file 1 [file anie0054-4764-sd1.pdf]

Angewandte Chemie
Eine Zeitschrift der Gesellschaft Deutscher Chemiker

Supporting Information

Target-Based Whole-Cell Screening by ^{1}H NMR Spectroscopy**

Junhe Ma, Qing Cao, Sarah M. McLeod, Keith Ferguson, Ning Gao, Alexander L. Breeze, and Jun Hu**

anie_201410701_sm_miscellaneous_information.pdf

CONTENTS:

1. Supporting figure and table

2. Materials and methods

2-1. Reagents

2-2. Percentage of inhibition

2-3. 96-well screening plate preparation

2-4. ^{1}H NMR measurements and analyses

1. Supporting figure and table

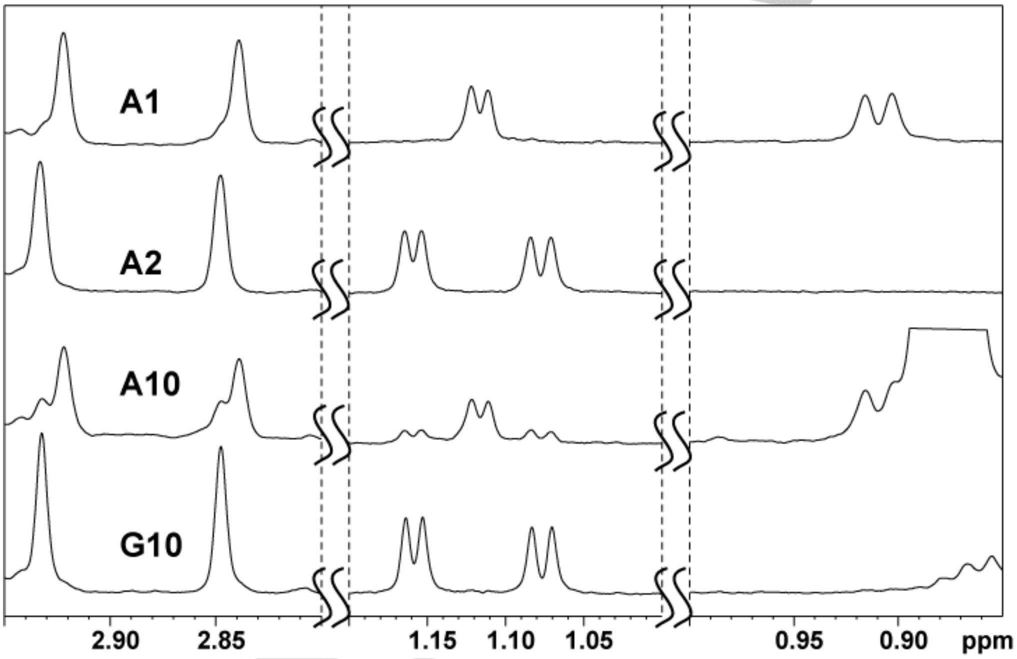

Figure S1. NMR signals of wells A1, A2 A10 and G10, showing the nitrogen attached methyl proton signals (2.95~2.80 ppm) and carbon attached methyl proton signals (1.20 ~ 1.00 ppm and 0.96~0.85 ppm).

[a] Dr. J. Ma, Q.Cao, N. Gao, Dr. J. Hu
Discovery Sciences, AstraZeneca Boston
[b] Dr. S. McLeod, K. Ferguson
Infection Innovative Medicines, AstraZeneca Boston
Waltham, Massachusetts 02451(USA)
E-mail: jun.hu2@astrazeneca.com
[c, d] Prof. A. L. Breeze
Astbury Centre for Structural Molecular Biology, Faculty of Biological Sciences, University of Leeds, Leeds, LS2 9JT, United Kingdom
A.L.Breeze@leeds.ac.uk

WILEY-VCH

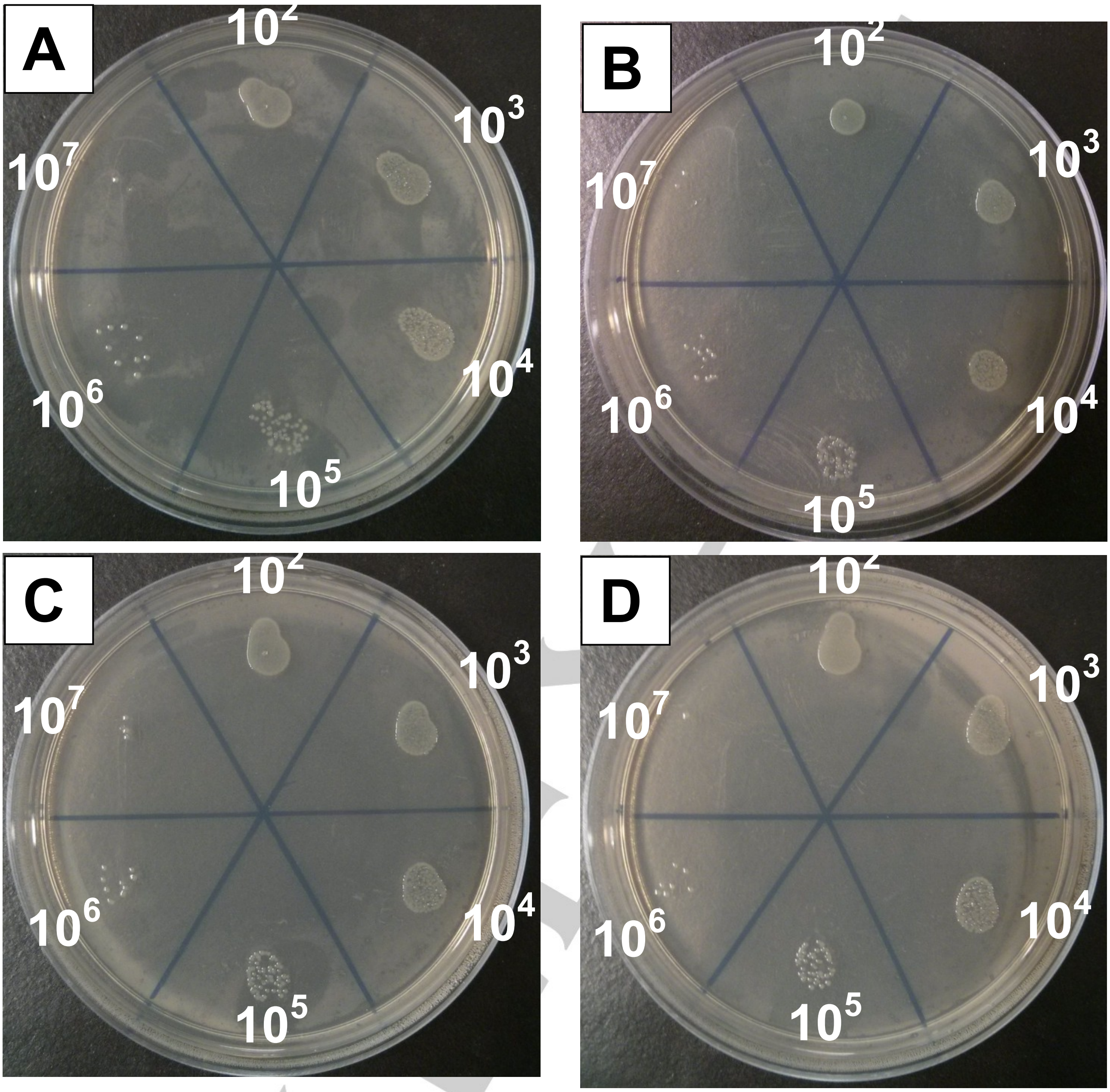
A
B
C
D
10^2
10^3
10^4
10^5
10^6
10^7

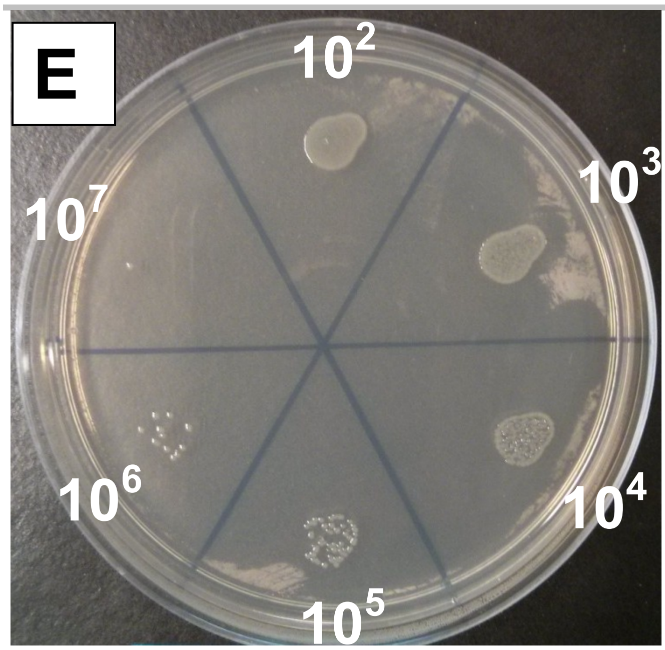

Figure S1. Plating colony tests to examine NDM-1 *E. coli* cell viability after treatment with screening compounds. All NDM-1 *E. coli* cells in these tests were from the same batch. The fold of dilution is labeled on each section of the LB/kanamycin plates. NDM-1 *E. coli* cells treated with DMSO were plated on plate A as reference. Cells with the addition of compounds from screening wells A5, B6 and C10 were plated on plate B, C and D, respectively. Cells with the addition of 5 different compounds (100 μM each) were plated on plate E. The section labeled with 10^6 fold dilution is used for the study of cell viability. There are 9, 11, 9, 8 and 11 single colonies on plate A, B C, D and E, respectively. The data demonstrate that cells were still alive after treatment with 0.5 mM screening compound or a combination of 5 compounds. The effects on cell viability after treatment with compounds are minimal.

WILEY-VCH

Table S1. Percentage of inhibition of screening compounds calculated based on the hydrolyzed meropenem signal intensity (same order as in the 96-well plate in Figure 3)

	1	2	3	4	5	6	7	8	9	10	11	12
**A**	0	99.9	2.0	9.3	73.0	1.2	23.0	63.2	64.4	-0.6	-0.8	99.9
**B**	25.1	86.9	7.1	8.7	7.0	93.0	90.2	11.6	-1.5	6.4	33.4	62.4
**C**	12.4	-2.0	9.0	12.1	12.9	6.1	6.2	39.3	11.3	31.1	37.0	58.3
**D**	99.9	-1.9	3.4	60.2	99.8	9.0	16.0	11.8	10.6	3.6	85.5	27.7
**E**	-1.0	99.9	5.2	4.8	1.3	2.0	81.0	0.8	1.2	-1.6	-1.7	4.0
**F**	-1.6	-4.1	-3.3	-1.1	-3.6	-0.9	-1.1	27.9	-0.8	-1.5	-0.4	-0.7
**G**	0.1	-3.1	-3.5	0.5	99.7	99.3	94.9	-1.3	80.2	98.9	90.3	95.2
**H**	-1.3	-0.6	99.2	2.9	-1.1	-2.1	-1.5	0.1	53.7	-0.8	99.5	-0.2

WILEY-VCH

2. Materials and methods

2-1. Reagents

Meropenem was purchased from Sigma-Aldrich Corporation. All inhibitors were either purchased from Sigma-Aldrich Corporation or from AstraZeneca in-house compound libraries. The compounds were dissolved in the deuterated DMSO as 100 mM stock solution.

The NDM-1 *Escherichia coli* cell strain was obtained following the previous protocol.[1]

2-2. Percentage of inhibition

Percentage of inhibition is calculated based on the intensity of product signal in the presence of compounds. The equation for the calculation is defined as following: [2]

Percentage inhibition = $(1- (I_0 – I_{EDTA}) / (I_{DMSO} - I_{EDTA})) \times 100\%$

where, I_0 is the intensity of the product signal in each well. I_{EDTA} is the intensity in the presence of EDTA. I_{DMSO} is the intensity in the presence of DMSO.

2-3. 96-well screening plate preparation

The 96-well plate for screening was prepared as following.
Step 1: 2.5 µl of 100 mM compounds, plus two d6-DMSO and EDTA controls, were deposited in each well of a 96-well plate.
Step 2: 275 µl fresh living NDM-1 *Escherichia coli* cells (OD_{600} = 0.25) in phosphate buffer (50 mM, pH 7.2) were added into the compound solution by multi-pipette and incubated at room temperature for at least half an hour.
Step 3: 10 µl of 5 mM meropenem was added into the mixture to allow the reaction to occur. In the mean time a NMR sample with the same condition of well A1 was prepared and put into the magnet to monitor the reaction.
Step 4: Once the reaction was half completed, 10 µl of 0.5 M EDTA was added to quench the reaction. After 30 minutes, the 96-well plate was transferred into a Bruker automatic Tecan/SampleRail system and each sample was mixed with 200 µl additional phoshpate buffer (50 mM, pH 7.2) before NMR measurement.

2-4. ^{1}H NMR measurements and analyses

All spectra were acquired on a Bruker 600MHz spectrometer with a TXI probe equipped with cryogenically cooled transmit/receive coils and a pulsed-field z-gradient coil. For 1D ^{1}H NMR experiments, a sweep width of 9615.385 Hz was used. Water suppression was achieved by means of the excitation sculpting scheme and the water-selective 180° Sinc shaped pulse was 3 ms long.[3] The free induction decay was collected in 32K data points. A 1 Hz line broadening function was applied during the Fourier transformation. All the spectra were acquired with a fixed number of scans of 64. The temperature was kept constant at 25 °C if not noted otherwise. NMR spectra were processed and analyzed with Topspin 3.2.

WILEY-VCH

References:

(1) J. Ma; S. McLeod, K. MacCormack, S. Sriram, N. Gao, A. L. Breeze, J. Hu, *Angew. Chem. Int. Ed. Engl.* **2014**, *53,* 2130-2133.

(2) M. E. Kovach, P. H. Elzer, D. S. Hill, G. T. Robertson, M. A. Farris, R. M. 2nd Roop, K. M. Peterson, *Gene* **1995**, *166*, 175-176.

(3) K. S. Scott, J. Keeler, T. L. Hwang, A. J. Shaka, *J. Am. Chem. Soc.* **1995**, *117*, 4199-4200.

WILEY-VCH
